# Should Expectations about the Rate of New Antiretroviral Drug Development Impact the Timing of HIV Treatment Initiation and Expectations about Treatment Benefits?

**DOI:** 10.1371/journal.pone.0098354

**Published:** 2014-06-25

**Authors:** Amin Khademi, R. Scott Braithwaite, Denis Saure, Andrew J. Schaefer, Kimberly Nucifora, Mark S. Roberts

**Affiliations:** 1 Department of Industrial Engineering, Clemson University, Clemson, South Carolina, United States of America; 2 Department of Medicine, New York University, New York City, New York, United States of America; 3 Division of Comparative Effectiveness and Decision Sciences, Department of Population Health, NYU School of Medicine, New York City, New York, United States of America; 4 Department of Industrial Engineering, University of Chile, Santiago, RM, Chile; 5 Department of Industrial Engineering, University of Pittsburgh, Pittsburgh, Pennsylvania, United States of America; 6 Department of Health Policy and Management, University of Pittsburgh Graduate School of Public Health, Pittsburgh, Pennsylvania, United States of America; 7 Department of Medicine, University of Pittsburgh School of Medicine, Pittsburgh, Pennsylvania, United States of America; Imperial College London, United Kingdom

## Abstract

**Background:**

Many analyses of HIV treatment decisions assume a fixed formulary of HIV drugs. However, new drugs are approved nearly twice a year, and the rate of availability of new drugs may affect treatment decisions, particularly when to initiate antiretroviral therapy (ART).

**Objectives:**

To determine the impact of considering the availability of new drugs on the optimal initiation criteria for ART and outcomes in patients with HIV/AIDS.

**Methods:**

We enhanced a previously described simulation model of the optimal time to initiate ART to incorporate the rate of availability of new antiviral drugs. We assumed that the future rate of availability of new drugs would be similar to the past rate of availability of new drugs, and we estimated the past rate by fitting a statistical model to actual HIV drug approval data from 1982–2010. We then tested whether or not the future availability of new drugs affected the model-predicted optimal time to initiate ART based on clinical outcomes, considering treatment initiation thresholds of 200, 350, and 500 cells/mm^3^. We also quantified the impact of the future availability of new drugs on life expectancy (LE) and quality-adjusted life expectancy (QALE).

**Results:**

In base case analysis, considering the availability of new drugs raised the optimal starting CD4 threshold for most patients to 500 cells/mm^3^. The predicted gains in outcomes due to availability of pipeline drugs were generally small (less than 1%), but for young patients with a high viral load could add as much as a 4.9% (1.73 years) increase in LE and a 8% (2.43 QALY) increase in QALE, because these patients were particularly likely to exhaust currently available ART regimens before they died. In sensitivity analysis, increasing the rate of availability of new drugs did not substantially alter the results. Lowering the toxicity of future ART drugs had greater potential to increase benefit for many patient groups, increasing QALE by as much as 10%.

**Conclusions:**

The future availability of new ART drugs without lower toxicity raises optimal treatment initiation for most patients, and improves clinical outcomes, especially for younger patients with higher viral loads. Reductions in toxicity of future ART drugs could impact optimal treatment initiation and improve clinical outcomes for all HIV patients.

## Introduction

The timing of HIV therapy initiation has been controversial, and remains so in resource-limited settings.[1], [2], [3], [4] In June 2013, the World Health Organization (WHO) updated its recommendation regarding the time to initiate therapy, now recommending an earlier initiation of ART when the CD4 count falls below 500 cells/ml. [5] Because timing decisions are not amenable to randomized controlled trials, this problem has been widely modeled and discussed in published reports.[6], [7], [8], [9], [10], [11] These models generally seek to identify the clinical conditions under which a patient should initiate ART so as to maximize his/her quality-adjusted life expectancy, and consider many factors, such as the initial viral load and CD4 count, age, gender, CD4 threshold and viral load threshold for initiating drugs, adherence, resistance, and HIV mutations at baseline. However, these models have not considered the rate of development of new antiretroviral drugs. In contrast, they have assumed fixed numbers of antiretroviral drugs assigned to a fixed number of distinct mechanistic categories, an unrealistic assumption because a pipeline of new ARTs is likely to continue. More ART options could make earlier initiation more favorable, because it could reduce the risk of accruing resistance to all available regimens. Additionally, newer ART regimens could be less toxic, also shifting the balance in favor of earlier ART. While ART initiation on detection has been advocated on the grounds of reducing the epidemic impact, this suggestion may not be persuasive for individuals who maximally value their health and well-being over those of the population at large.

Accordingly, we developed a model to address how ART initiation recommendations would change with varying assumptions regarding the rates of new drug development, the proportion of new drugs in existing classes versus new mechanistic classes, the patterns of cross-resistance between new and existing mechanistic classes, the efficacy of pipeline drugs compared to existing drugs, and the toxicity of new drugs compared to existing drugs.

## Methods

We adapted the well-validated HIV simulation model of Braithwaite et al.[6] to consider different assumptions regarding the availability and characteristics of new antiretroviral drugs. The Braithwaite model has been described in detail elsewhere,[6], [12], [13] but will be briefly described here. A graphical representation with further explanation is provided in Appendix S1. The model is an individual microsimulation that tracks the individual progression of disease (CD4 counts, viral loads, presence of mutations, treatment status, etc.) and estimates HIV-related mortality as a function of those individual patient characteristics.[12] It estimates baseline non-HIV mortality as a function of age and gender, HIV-related mortality is a function of CD4 count, and mortality is also affected by the toxicity of ARVs. The rate of decline in CD4 count is a function of the current VL, the presence of treatment and demographic factors. A notable aspect of the model is the mechanistic manner by which the model represents the development of HIV antiviral resistance.[12], [13], [14] Each individual in the model has a simplistic representation of the viral genome which mutates as a function of replication rate, and a mutation becomes established in the population only of a mutation occurs to a drug that the patient is on, providing selection pressure. Effective ART therapy decreases the replication rate, which decreases the mutation rate. Once a mutation becomes established, the replication rate and VL increase, and the CD4 count declines. The model has been demonstrated to predict the time to treatment failure, survival, and the development of HIV antiviral resistance.[6], [13], [15]

We modified this model by incorporating the arrival of new drugs, both within existing classes of drugs and the development of new classes of antiretroviral agents. This modification allows the simulation to treat patients with more cycles of therapy, and provides increased flexibility for changing to a different drug combination after the development of resistance.

In our base case, we simulated a cohort of patients treated under the assumption of the availability of three classes of antiretroviral drugs, without the future development of new drugs, which is a common assumption used by most HIV treatment models.[1], [6] We then compared the life expectancy and quality-adjusted life expectancy of an identical cohort treated under alternative scenarios that assumed new ART drugs would become available, seeking to estimate how this ART pipeline would influence the optimal criteria for ART initiation. Because of considerable uncertainty regarding the effectiveness and toxicity of new drugs, we explored a wide variety of assumptions. However, we centered these assumptions on historical data describing the arrival rate of ART drugs for both existing and new classes of drugs, and the likelihood of cross-resistance between new and existing mechanistic classes.

### Estimating the arrival rate of new ART drugs

We fit the probability distribution for the arrival of new ART drugs, defined as when the drug was approved by the Food and Drug Administration (FDA), to an exponential distribution using data from the FDA of the approval date of each drug and each new drug class (see Table A1 and A2 in Appendix S1).[16] The parameters of distributions were estimated by the Maximum Likelihood Estimator technique. Goodness of fit was tested by Quantile-Quantile plot and Kolmogorov-Smirnov tests.[17] The statistical programming language “R” was used for all estimates and statistical tests.

**Figure 1:**
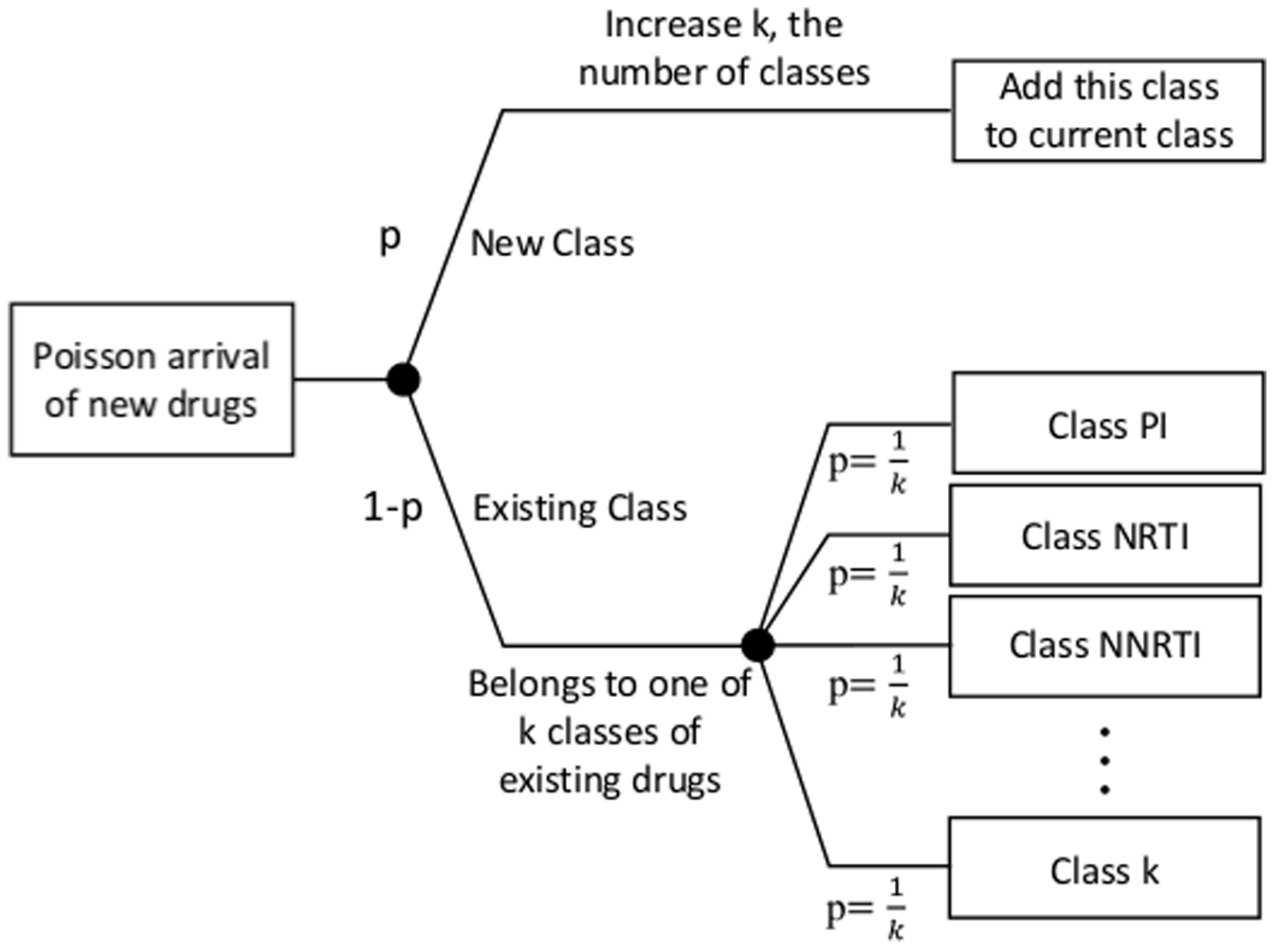
Arrival process of pipeline drugs. The arrival process of HIV pipeline drugs follows a split Poisson process. See text for details.

An exponential inter-arrival time implies that the number of new events in a given time will follow a Poisson process. We assumed that the new drug arrival was a combination of two processes: 1) the arrival process of new classes of drugs, and 2) the arrival process of new drugs belonging to existing classes. We fit the arrival process of drugs to a split Poisson process, meaning that the arrival of new classes is independent from the arrival processes of drugs within an existing class.[18] Moreover, if a new drug arrived belonging to an existing class, we assumed that it will be uniformly distributed among current classes. A schematic view is shown in Figure 1. See Table A1 in Appendix S1.

### Estimating Cross-resistance of new antiretroviral agents

The development of HIV antiviral resistance is complex, and mutations in the HIV genome that confer resistance to a particular drug may also confer partial resistance to other drugs. Since cross resistance may affect new drugs as it does existing ones, we modeled the probability that new drugs would confer cross resistance. Empirically, cross resistance is substantially more likely in drugs within the same class than between drugs of a different class.[19] For example, mutations in the NRTI and NNRTI class are both in the Reverse Transcriptase gene, but there is no mutual mutation between these classes, so there is no cross-resistance pattern between the NRTI and NNRTI classes. Since mutations in the PI class occur in the Protease gene and in the NRTI and NNRTI class occur in Reverse Transcriptase gene,[19] there is no cross-resistance between these classes either.

The cross resistance of new drugs within a class is assumed to be equal to the distribution of cross-resistance patterns of existing drugs, i.e., if a new drug belongs to the NRTI class, it will follow the resistance pattern of the NRTI class. The probability that a specific mutation will confer resistance to a new drug is estimated by the proportion of drugs in that class for which the mutation is known to confer resistance. For example, in the NRTI class, the M41 mutation confers resistance only to Stavudine and Zidovudine, so the total number of drugs for which M41 confers resistance is 2 out of 7 drugs.[19] This procedure was repeated for all mutations in each class. Then, the best-fitting probability distribution was determined based on the number of drugs to which each mutation confers resistance.

### The efficacy and toxicity of new antiretroviral agents

The efficacy of a particular drug is represented by its ability to decrease viral load under perfect adherence. Our baseline assumption is that efficacy of new drugs (the viral load decrement at perfect adherence) is equal to the average of viral load decrements observed by drugs in the same class as the new drug, under scenarios of near-perfect adherence. In our base case analysis, we assume that the toxicity of new drugs was similar to the toxicity level of existing drugs within that category. However, in sensitivity analysis we explore scenarios in which the efficacy and toxicity of pipeline drugs are different from existing drugs.

### Scenarios regarding when to initiate ART

Successive populations of individuals with newly diagnosed chronic HIV infection were considered, each of them starting with CD4 count of 500 cells/mm^3^. We considered alternative CD4 thresholds for ART initiation varying from 50 cells/mm^3^ to 500 cells/mm^3^, in increments of 50 cells/mm^3^. In addition, different starting age categories (30, 40, and 50) and baseline viral loads of 10^4^, 10^4.5^, 10^5^ and 10^5.5^ copies/mL were modeled. In each scenario, the optimal CD4 count at which to initiate treatment was identified by finding the CD4 count (up to 500 cell/mm^3^) that produced the maximum life expectancy. Additionally, in each scenario, we compared increases in life expectancy (LE) and quality-adjusted life expectancy (QALE) that could arise from the expected rate of development of new ARTs.

### Sensitivity analyses

We varied several assumptions to assess robustness of model predictions, particularly regarding the pipeline of new ART drugs. Specifically, we varied the inter-arrival time of new drugs, the rate at which new drugs induce resistance mutations, the propensity to adhere with ART overall, the effectiveness of new pipeline drugs compared to existing drugs, and the toxicity level of new pipeline drugs compared to existing drugs. In scenarios in which the efficacy of the new drug is equivalent to the average of the existing drugs, the new drug is added as an extra regimen when the patient has exhausted all existing regimens. However, in sensitivity analysis where the new drug is more effective than existing drugs, we assume the new drug is used after the first regimen has failed.

## Results

### The arrival rate of new ART drugs

The distribution of inter-arrival times of drugs was satisfied by assuming an exponential distribution with a mean inter-arrival time of 7. 69 months (e.g. mean 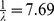 months, which implies that the arrival of new drugs follows a Poisson process with rate 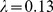; quantitle-quantile plot shown in Figure S3 of Appendix S1). When a new drug arrives, its arrival time was fit by a split Poisson process, meaning that it would be from a new class with probability 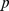 (which we estimated at 0.194) and from current classes with probability 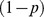 (which we estimated at 0.806). The Kolmogorov-Smirnov (KS) goodness of fit test associated with these distributions is shown in Table 1.

**Table 1:** Inter-arrival time distributions.

	Probability distribution	P-value	95% CI
Inter-arrival time of new drugs	Exponential ( 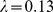 )	0.085	[0.09,0.181]
Inter-arrival time of new classes	Exponential ( 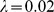 )	0.725	[0.006,0.041]
Inter-arrival time of new drugs belonging to existing classes	Exponential ( 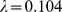 )	0.112	[0.068,0.147]

A Poisson process produces exponential inter-arrival distribution.

### The distribution of new drugs between existing classes and new classes

Between 21 June 1996 and 13 March 2003 there was 3 classes of ART drugs, and 11 new drugs became available, of which 4 belonged to the PI class, 5 belonged to the NRTI class and 2 belonged to the NNRTI class. Fitting a uniform distribution to this data has the p-value = 0.08253, supporting the assumption that new drugs are distributed equally among existing classes.

### The cross resistance rates of new drugs

The best fitting distributions for the probability of cross resistance within NRTIs and NNRTs were also uniform, whereas the probability of cross resistance within PIs was best fit by a Poisson distribution (Table 2). The cross-resistance probability was estimated at 0.2 between TAM and Non-TAM variants of NRTIs. Note that for Fusion Inhibitors, Entry Inhibitors, and Integrase Inhibitors no distribution could be fit since there was only one drug in each class at the time of analysis. Although the KS tests for these distributions have a p-value that indicates the empiric distribution is statistically different from the estimated distribution, these are the distributions with the “best” fit – no other distribution had a larger p-value.

**Table 2:** Resistance distributions for existing drug classes.

	Drug class		
	NRTI	NNRTI	PI
Number of drugs	7	3	8
Probability distribution of number of drugs resistant to a mutation	Uniform[1], [4] (p-value* = 0.059)	Uniform[1], [3] (p-value = 0.042)	Poisson( 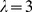 ) (p-value = 0.024)

* p-value is for the for Kolmogorov-Smirnov goodness of fit test.

### The effect of new ART drug arrival on time to initiate therapy and clinical outcomes

**Figure 2:**
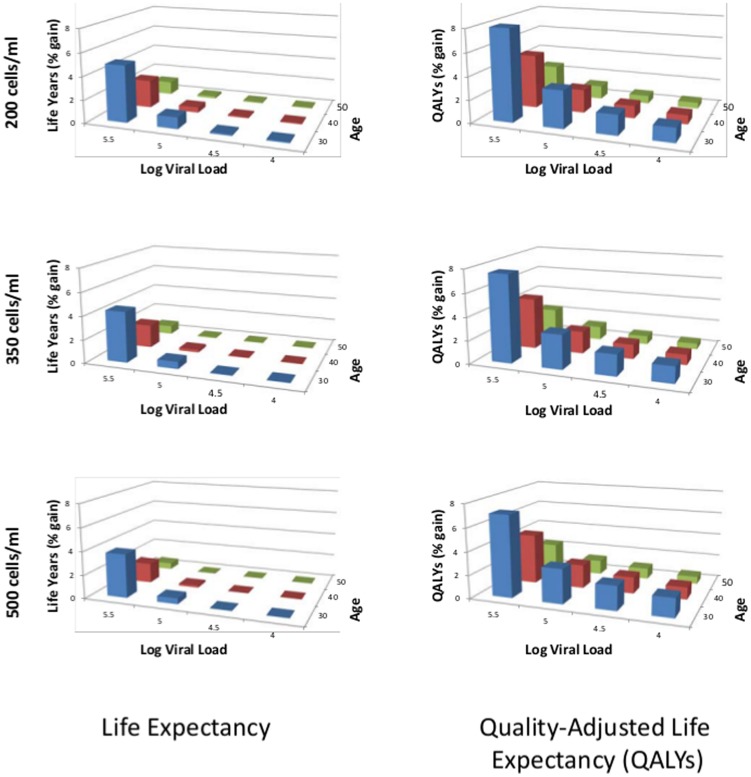
Percent change in outcomes from the presence of pipeline drugs by age, viral load, and CD4 count at initiation of therapy. The graphs on the left depict the percent change in life expectancy from the presence of pipeline drugs, the graphs on the right the percent change in quality-adjusted life expectancy.

Table 3 shows the optimal CD4 count threshold for initiating ART, comparing scenarios with versus without the rate of accrual of new ART drugs that we estimated above. Simulations with and without pipeline drugs suggested that treatment at a CD4 count of 500 cells/mm^3^ was preferred for all patients except for those with advanced age and/or low viral load which is consistent with the new WHO guidelines. For these patient subgroups, earlier initiation was only preferred when considering the likely ART pipeline, whereas later initiation was preferred when considering only the formulary of currently available drugs. In general, the effects of the availability of pipeline drugs are small (Figure 2). New ART drugs are most likely to add health benefits for younger individuals with higher viral loads, which is intuitive because these individuals are more likely to “burn through” existing regimens before they die of non-HIV-related causes. Indeed, the availability of pipeline drugs added as much as 4.9% to life expectancy (8.0% to QALE) for 30 year-olds with the highest viral loads (>5.5 log) for therapy initiated late (starting at 200 cells/mm^3^). However, the life expectancy gains to pipeline drugs for most patients remain less than 1%.

**Table 3:** The optimal CD4 count threshold for initiating therapy.

	Age 30 years		Age 40 years		Age 50 years	
Log VL	No Pipeline	Pipeline	No Pipeline	Pipeline	No Pipeline	Pipeline
4.0	450	500	350	500	350	450
4.5	450	500	500	500	450	500
5.0	450	500	500	500	450	500
5.5	450	500	500	500	500	500

### Sensitivity analyses

**Figure 3:**
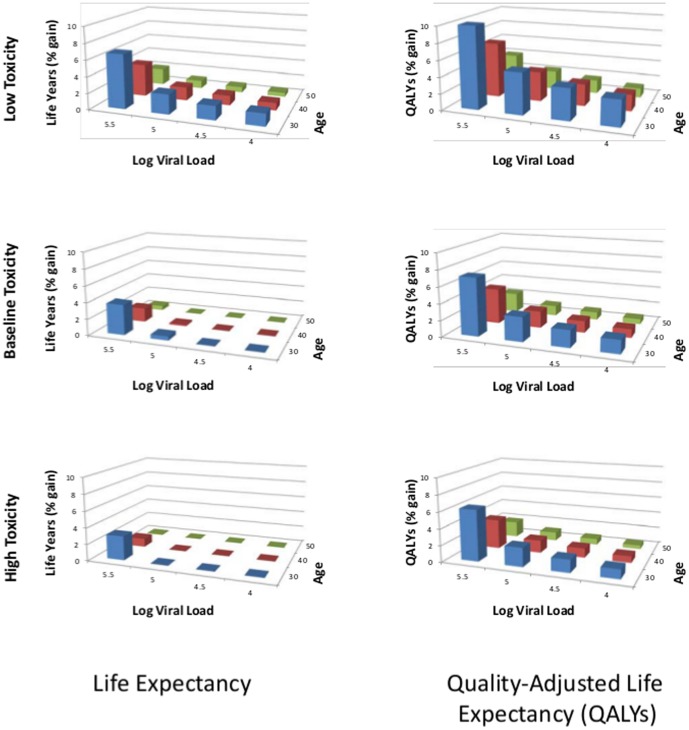
Sensitivity analysis of effect percent change in LE and QALE from the presence of pipeline drugs by age, viral load, and toxicity. The graphs on the left depict the percent change in life expectancy assuming new drugs have a lower toxicity than existing drugs (top), identical toxicity to existing drugs (middle) or a higher toxicity that existing drugs (bottom). The graphs on the right depict the percent change in quality-adjusted life expectancy for the same toxicity levels.

Our sensitivity analyses (Table 4) indicate that varying the estimates of the inter-arrival times of new drugs, the rate of accumulation of resistance, the patient's adherence to treatment regimens, and the relative efficacy of pipeline drugs have little effect on overall outcomes, but that the toxicity of pipeline drugs has a potentially large effect on life expectancy. If the toxicity of pipeline drugs is reduced compared to existing drugs (the pipeline drugs have a mortality relative risk of 1), the presence of new pipeline drugs can increase the quality-adjusted life expectancy by as much as 11% in young patients with high viral load. Figure 3 shows the change in LE and QALE (in percent) due to the presence of pipeline drugs for a variety of toxicity, age, and viral load category. Aligned with the new WHO recommendation we consider the CD4 threshold of 500 cells/mL for treatment initiation.

**Table 4:** Sensitivity analysis of pipeline effect with varying different parameter assumptions.

**Age 30 years**								
				**VL**	4.0	4.5	5.0	5.5
	**Low**	**Base**	**High**					
**Inter-arrival time of pipeline drugs (according to 95% confidence interval)**	0.09	0.13	0.181		(1.77,1.64,1.75)	(2.28,2.09,2.02)	(2.87,2.99,3.86)	(6.97,7.07,7.47)
**Rate of accumulating resistance mutations of pipeline drugs**	0.16	0.18	0.2		(1.78,1.64,1.34)	(2.01,2.09,1.94)	(3.59,2.99,2.95)	(7.29,7.07,7.24)
**Adherence (proportion of medication doses taken as directed)**	0.62	0.75	0.76		(1.12,1.64,1.75)	(1.08,2.09,2.01)	(2.51,2.99,3.58)	(6.08,7.07,7.26)
**Augmentation in viral load decrement of pipeline drugs, additive (log units)**	−1	0	1		(1.75,1.64,1.75)	(1.88,2.09,2.02)	(3.58,2.99,3.58)	(7.25,7.07,7.36)
**Toxicity of pipeline drugs**	1	1.5	1.5		(3.17,1.64,1.13)	(3.84,2.09,1.56)	(5.05,2.99,2.31)	(10.23,7.07,6.22)
**Age 40 years**								
				**VL**	4.0	4.5	5.0	5.5
	**Low**	**Base**	**High**					
**Inter-arrival time of pipeline drugs (according to 95% confidence interval)**	0.09	0.13	0.181		(1.03,1.06,0.86)	(1.46,1.44,1.70)	(1.92,2.06,2.58)	(4.10,4.32,4.87)
**Rate of accumulating resistance mutations of pipeline drugs**	0.16	0.18	0.2		(0.86,1.06,0.53)	(1.70,1.44,1.70)	(1.86,2.06,1.86)	(4.32,4.32,4.73)
**Adherence (proportion of medication doses taken as directed)**	0.62	0.75	0.76		(0.74,1.06,0.86)	(1.00,1.44,1.70)	(1.16,2.06,1.95)	(3.52,4.32,4.86)
**Augmentation in viral load decrement of pipeline drugs, additive (log units)**	−1	0	1		(0.86,1.06,0.86)	(1.6,1.44,1.70)	(1.86,2.06,1.94)	(4.58,4.32,4.86)
**Toxicity of pipeline drugs**	1	1.5	1.7		(2.02,1.06,0.8)	(2.7,1.44,1.15)	(3.62,2.06,1.54)	(6.78,4.32,3.57)
**Age 50 years**								
				**VL**	4.0	4.5	5.0	5.5
	**Low**	**Base**	**High**					
**Inter-arrival time of pipeline drugs (according to 95% confidence interval)**	0.09	0.13	0.181		(0.80,0.64,0.13)	(1.00,0.95,0.71)	(1.22,1.26,1.55)	(2.17,2.38,2.18)
**Rate of accumulating resistance mutations of pipeline drugs**	0.16	0.18	0.2		(0.52,0.64,0.05)	(1.55,0.95,0.97)	(1.55,1.26,0.78)	(2.57,2.38,1.75)
**Adherence (proportion of medication doses taken as directed)**	0.62	0.75	0.76		(0.00,0.64,0.12)	(0.44,0.95,1.55)	(0.30,1.26,1.55)	(1.4,2.38,2.08)
**Augmentation in viral load decrement of pipeline drugs, additive (log units)**	−1	0	1		(0.05,0.64,0.05)	(1.55,0.95,1.55)	(1.43,1.26,1.55)	(2.08,2.38,2.56)
**Toxicity of pipeline drugs**	1	1.5	1.7		(1.15,0.64,0.46)	(1.66,0.95,0.73)	(2.3,1.26,1.06)	(3.99,2.38,1.9)

The numbers are the QALYs gain percentage due to pipeline drugs. Starting CD4 count for all categories is 500 cells/mL

## Discussion

Consistent with current treatment recommendations, the base case model (that does not include the availability of pipeline drugs) supports early treatment in most scenarios. The addition of pipeline drugs raises the CD4 threshold for treatment in several classes of patients, resulting in the optimal CD4 to initiate treatment across virtually all ages and viral loads to be 500 cells/mm^3^, which supports the current WHO recommendations. However, the overall impact of a pipeline of new drugs is not large for most patient groups, with average changes in life expectancy less that 1%, except for patients who are young and have high viral loads, primarily because there are already a large number of regimens available. Our results are supported by intuition, which would suggest that the availability of more drugs would reduce the likelihood of “burning through” existing regimens for patients at highest risk for doing so, in particular patients with higher baseline viral loads.

Our analysis does indicate that patients will be better served if new HIV drugs are found with lower side effects and toxicities: this remains one of the most important reasons that drug regimens are discontinued, and decreased toxicities and side effects will extend the duration of the regimen.

This study has several limitations. Our data are from a cohort that is overwhelmingly male, and thus our results may not apply for women. Cost or cost-effectiveness has not been considered in our analyses, which may have impact on policy recommendations. Our model does not include spreading of resistance patterns in the native viral population. As resistance spreads, some newly infected individuals may be infected with already resistant (non- “wild type”) strains. In addition, our analysis does not consider the impact of pipeline drugs on the epidemic – the model does not represent transmission between individuals. To the extent that new pipeline drugs will lower the VL of some individuals, it would be expected to decrease transmission. However, because the addition of pipeline drugs extends the ability to have effective treatment towards the end of a patient's disease, the effect is expected to be small. Finally, we did not perform subgroup analyses for patient groups likely to have poor adherence (e.g. persons with substance abuse, unhealthy alcohol use, or mental illness), who might be more likely to exhaust existing regimens because of resistance or intolerance, and who therefore would yield disproportional advantages from the development of new drugs. In summary, our results suggest that the rate of development of new ART drugs may not impact the starting threshold for most patient groups, although they may substantially increase benefit for younger patients with higher viral loads. Finally, our sensitivity analyses raise the intriguing prospect that reducing the toxicity profile of new ART drugs may have a greater beneficial impact on health than increasing the supply of new drugs with novel mechanisms or resistance patterns.

## Supporting Information

Appendix S1 Contains Figure S1, Basic structure of HIV simulation model. See text for details. Figure S2, Typical patient histories with and without pipeline drugs. See text for details. Figure S3, The Quantile-Quantile plot for pipeline arrival process. Quantile-Quantile plots are used to compare a dataset to a theoretical distribution. It provides an assessment of graphical goodness of fit. If the points lie on the line, the probability distribution is acceptable.
(DOCX)
