# Correction: Should Expectations about the Rate of New Antiretroviral Drug Development Impact the Timing of HIV Treatment Initiation and Expectations about Treatment Benefits?

**DOI:** 10.1371/journal.pone.0108643

**Published:** 2014-09-12

**Authors:** The PLOS ONE Staff

Figures S1, S2 and S3 are omitted from the Appendix S1 file in the Supporting Information. They can be viewed below.

## Supporting Information

Figure S1 **Basic structure of HIV simulation model.**See text for details.
(TIF)

Figure S2 **Typical patient histories with and without pipeline drugs.** See text for details.
(TIF)

Figure S3 **The Quantile-Quantile plot for pipeline arrival process.** Quantile-Quantile plots are used to compare a dataset to a theoretical distribution. It provides an assessment of graphical goodness of fit. If the points lie on the line, the probability distribution is acceptable.
(TIF)
