# Supplementary material for: Should Expectations about the Rate of New Antiretroviral Drug Development Impact the Timing of HIV Treatment Initiation and Expectations about Treatment Benefits?
Source: PLoS One. 2014 Jun 25;9(6):e98354. doi: 10.1371/journal.pone.0098354 (PMC4070901; doi:10.1371/journal.pone.0098354)
Supplement: Appendix S1 — Contains Figure S1, Basic structure of HIV simulation model. See text for details. Figure S2, Typical patient histories with and without pipeline drugs. See text for details. Figure S3, The Quantile-Quantile plot for pipeline arrival process. Quantile-Quantile plots are used to compare a dataset to a theoretical distribution. It provides an assessment of graphical goodness of fit. If the points lie on the line, the probability distribution is acceptable. (DOCX) [file pone.0098354.s001.docx]

**APPENDIX: Should expectations about the rate of new antiretroviral drug development impact the timing of HIV treatment initiation and expectations about treatment benefits?**

**Description of the Braithwaite HIV Microsimulation Model**

The HIV simulation model is an individual microsimulation model in which each virtual individual is tracked through the course of their disease. It can assign otherwise similar patient cohorts to different treatment decisions and compare the effect of this decision on designated outcomes. Because the simulation is probabilistic, it can represent much of the heterogeneity of actual patient populations (for example, clinical events, such as death, may or may not happen within any particular time interval, and their probability of occurrence is based on known predictors). A patient is initialized with several characteristics including age, gender, CD4 count, and viral load, which is related to a viral load set point that has a distribution in the population. A schematic of the relationships represented in the model is shown in Figure A1. Each individual in the model has their own copy of the simulation model that determines the progression of disease. The model is described in detail in several references [^1-5^](#_ENREF_1) but the mechanics will be briefly described here.

The progression of disease occurs through estimating the decrement in CD4 count in a given time. These functions are statistically derived from large data sets and predict the fall in CD4 count as a function of viral load, the presence of treatment and many other clinical variables. Viral load is determined as a function of the set point and the presence or absence of treatment. As the virus replicates, it mutates proportional to the replication rate. Mutations to the various classes of antiretroviral medications occur at rates derived from the literature.[^6^](#_ENREF_6) The presence of antiretroviral therapy decreases replication which decreases viral load. The efficacy of antiretroviral medications in decreasing the viral load was estimated from multiple randomized controlled trials and studies in the literature, which are referenced in several background papers[^1^](#_ENREF_1)^,^ [^4^](#_ENREF_4)^,^ [^5^](#_ENREF_5) and will not be repeated here.

As the patient progresses, death from HIV disease is a statistical function of the CD4 count and other clinical characteristics; death from other causes is a function of the patient’s clinical and demographic characteristics. These functions can be made arbitrarily complex, however for the purpose of this simulation death from other causes is only a function of age, gender and the toxicity of antiretroviral drugs.

A unique aspect of the simulation model is the mechanism by which resistance to antiretroviral therapy appears. Since the virus in each individual is replicating at a certain rate, and each class of mutations occurs at a certain rate that is a function of the baseline replication rate, a mutation to a specific antiretroviral agent only appears and takes hold in that individual if two events occur: 1) the patient develops a mutation to a specific drug class, and 2) the patient is on a drug of that class, which provides the selection pressure. Therefore it is the combination of a specific mutation and the presence of selection pressure by being on that drug that produces antiretroviral resistance. This mechanism has been shown to be effective at predicting the rate of accumulation of antiretroviral resistance, as well as time to treatment failure.[^3-5^](#_ENREF_3)

Figure A2, panel A illustrates a typical disease history for a patient in the model. The patient is initiated with a specific VL and CD4 count, and therapy is started or not based on a treatment algorithm. The CD4 count and VL are estimated over time, and treatment changes are made when certain criteria for treatment failure are reached. As all potential regimens are exhausted, the patient’s VL rises to its set point, the CD4 count falls, and eventually the patient dies.

**Addition of New Pipeline” Medications**

The standard simulation contains the current set of medications available (by class and instance within class). Once a patient develops a resistance to a regimen, and algorithm in the program switches the patient to a different regimen. Regimens can be switched because of two reasons: 1) the development of antiretroviral resistance, or 2) the development of intolerance to side effects. When the patient has gone through all possible regimens, the patient remains on that regimen even after the development of resistance until the patient dies. Figure A2 describes the mechanism by which we compare the presence of pipeline drugs to the absence of new pipeline drugs. The top panel illustrates the progression of one individual through the model in the absence of the availability of new drugs. The patient’s viral load is suppressed initially by the presence of antiretroviral therapy, and then a mutation develops to a medicine that the patient is on, the regimen is changed, another mutation eventually develops, again the regimen is changed, and this will happen until all of the potential regimens are exhausted. In this illustration we show for potential regimens. The lower panel demonstrates the same person’s history in the presence of pipeline drugs, which allow in this case one more regimen because of the appearance of one more medication during the lifespan of this patient. In this particular case the patient lives for a longer period of time given the improvement in CD4 count seen by the presence of a new extra regimen. The simulation compares the outcomes across many thousands of people whose outcomes are evaluated under the scenario of no pipeline drugs and compares that to their life expectancies and other outcome variables in the presence of new pipeline drugs.

**Table A1: Approval date of each new class of drug**

| **Drug class** | **Date of approval** |
| --- | --- |
| NRTI | 19 March 1987 |
| PI | 6 Dec 1995 |
| NNRTI | 21 June 1996 |
| Fusion Inhibitor | 13 March 2003 |
| CCR5 | 6 August 2007 |
| Integrase Inhibitors | 12 October 2007 |

**Table A2: Approval dates of all HIV drugs**

**Nucleoside Reverse Transcriptase Inhibitors (NRTIs)**

| **Brand Name** | **Generic Name** | **Manufacturer Name*** | **Approval Date** | **Time to Approval** |
| --- | --- | --- | --- | --- |
| [Combivir](http://www.accessdata.fda.gov/scripts/cder/drugsatfda/index.cfm?fuseaction=Search.SearchAction&SearchTerm=Combivir&SearchType=BasicSearch) | lamivudine and zidovudine | GlaxoSmithKline | 27-Sep-97 | 3.9 months |
| [Emtriva](http://www.accessdata.fda.gov/scripts/cder/drugsatfda/index.cfm?fuseaction=Search.SearchAction&SearchTerm=Emtriva&SearchType=BasicSearch) | emtricitabine, FTC | Gilead Sciences | 02-Jul-03 | 10 months |
| [Epivir](http://www.accessdata.fda.gov/scripts/cder/drugsatfda/index.cfm?fuseaction=Search.SearchAction&SearchTerm=Epivir&SearchType=BasicSearch) | lamivudine, 3TC | GlaxoSmithKline | 17-Nov-95 | 4.4 months |
| [Epzicom](http://www.accessdata.fda.gov/scripts/cder/drugsatfda/index.cfm?fuseaction=Search.SearchAction&SearchTerm=Epzicom&SearchType=BasicSearch) | abacavir and lamivudine | GlaxoSmithKline | 02-Aug-04 | 10 months |
| [Hivid](http://www.accessdata.fda.gov/scripts/cder/drugsatfda/index.cfm?fuseaction=Search.SearchAction&SearchTerm=Hivid&SearchType=BasicSearch) | zalcitabine, dideoxycytidine, ddC (no longer marketed) | Hoffmann-La Roche | 19-Jun-92 | 7.6 months |
| [Retrovir](http://www.accessdata.fda.gov/scripts/cder/drugsatfda/index.cfm?fuseaction=Search.SearchAction&SearchTerm=Retrovir&SearchType=BasicSearch) | zidovudine, azidothymidine, AZT, ZDV | GlaxoSmithKline | 19-Mar-87 | 3.5 months |
| [Trizivir](http://www.accessdata.fda.gov/scripts/cder/drugsatfda/index.cfm?fuseaction=Search.SearchAction&SearchTerm=Trizivir&SearchType=BasicSearch) | abacavir, zidovudine, and lamivudine | GlaxoSmithKline | 14-Nov-00 | 10.9 months |
| [Truvada](http://www.accessdata.fda.gov/scripts/cder/drugsatfda/index.cfm?fuseaction=Search.SearchAction&SearchTerm=Truvada&SearchType=BasicSearch) | tenofovir disoproxil fumarate and emtricitabine | Gilead Sciences, Inc. | 02-Aug-04 | 5 months |
| [Videx EC](http://www.accessdata.fda.gov/scripts/cder/drugsatfda/index.cfm?fuseaction=Search.SearchAction&SearchTerm=Videx%20EC&SearchType=BasicSearch) | enteric coated didanosine, ddI EC | Bristol Myers-Squibb | 31-Oct-00 | 9 months |
| [Videx](http://www.accessdata.fda.gov/scripts/cder/drugsatfda/index.cfm?fuseaction=Search.SearchAction&SearchTerm=Videx&SearchType=BasicSearch) | didanosine, dideoxyinosine, ddI | Bristol Myers-Squibb | 9-Oct-91 | 6 months |
| [Viread](http://www.accessdata.fda.gov/scripts/cder/drugsatfda/index.cfm?fuseaction=Search.SearchAction&SearchTerm=Viread&SearchType=BasicSearch) | tenofovir disoproxil fumarate, TDF | Gilead | 26-Oct-01 | 5.9 months |
| [Zerit](http://www.accessdata.fda.gov/scripts/cder/drugsatfda/index.cfm?fuseaction=Search.SearchAction&SearchTerm=Zerit&SearchType=BasicSearch) | stavudine, d4T | Bristol Myers-Squibb | 24-Jun-94 | 5.9 months |
| [Ziagen](http://www.accessdata.fda.gov/scripts/cder/drugsatfda/index.cfm?fuseaction=Search.SearchAction&SearchTerm=Ziagen&SearchType=BasicSearch) | abacavir sulfate, ABC | GlaxoSmithKline | 17-Dec-98 | 5.8 months |

**Nonnucleoside Reverse Transcriptase Inhibitors (NNRTIs)**

| **Brand Name** | **Generic Name** | **Manufacturer Name*** | **Approval Date** | **Time to Approval** |
| --- | --- | --- | --- | --- |
| [Intelence](http://www.accessdata.fda.gov/scripts/cder/drugsatfda/index.cfm?fuseaction=Search.SearchAction&SearchTerm=Intelence&SearchType=BasicSearch) | etravirine | Tibotec Therapeutics | 18-Jan-08 | 6 months |
| [Rescriptor](http://www.accessdata.fda.gov/scripts/cder/drugsatfda/index.cfm?fuseaction=Search.SearchAction&SearchTerm=Rescriptor&SearchType=BasicSearch) | delavirdine, DLV | Pfizer | 4-Apr-97 | 8.7 months |
| [Sustiva](http://www.accessdata.fda.gov/scripts/cder/drugsatfda/index.cfm?fuseaction=Search.SearchAction&SearchTerm=Sustiva&SearchType=BasicSearch) | efavirenz, EFV | Bristol Myers-Squibb | 17-Sep-98 | 3.2 months |
| [Viramune](http://www.accessdata.fda.gov/scripts/cder/drugsatfda/index.cfm?fuseaction=Search.SearchAction&SearchTerm=Viramune&SearchType=BasicSearch)  (Immediate Release) | nevirapine, NVP | Boehringer Ingelheim | 21-Jun-96 | 3.9 months |

**Protease Inhibitors (PIs)**

| **Brand Name** | **Generic Name** | **Manufacturer Name*** | **Approval Date** | **Time to Approval** |
| --- | --- | --- | --- | --- |
| [Agenerase](http://www.accessdata.fda.gov/scripts/cder/drugsatfda/index.cfm?fuseaction=Search.SearchAction&SearchTerm=Agenerase&SearchType=BasicSearch) | amprenavir, APV (no longer marketed) | GlaxoSmithKline | 15-Apr-99 | 6 months |
| [Aptivus](http://www.accessdata.fda.gov/scripts/cder/drugsatfda/index.cfm?fuseaction=Search.SearchAction&SearchTerm=Aptivus&SearchType=BasicSearch) | tipranavir, TPV | Boehringer Ingelheim | 22-Jun-05 | 6 months |
| [Crixivan](http://www.accessdata.fda.gov/scripts/cder/drugsatfda/index.cfm?fuseaction=Search.SearchAction&SearchTerm=Crixivan&SearchType=BasicSearch) | indinavir, IDV, | Merck | 13-Mar-96 | 1.4 months |
| [Fortovase](http://www.accessdata.fda.gov/scripts/cder/drugsatfda/index.cfm?fuseaction=Search.SearchAction&SearchTerm=Fortovase&SearchType=BasicSearch) | saquinavir (no longer marketed) | Hoffmann-La Roche | 7-Nov-97 | 5.9 months |
| [Invirase](http://www.accessdata.fda.gov/scripts/cder/drugsatfda/index.cfm?fuseaction=Search.SearchAction&SearchTerm=Invirase&SearchType=BasicSearch) | saquinavir mesylate, SQV | Hoffmann-La Roche | 6-Dec-95 | 3.2 months |
| [Kaletra](http://www.accessdata.fda.gov/scripts/cder/drugsatfda/index.cfm?fuseaction=Search.SearchAction&SearchTerm=Kaletra&SearchType=BasicSearch) | lopinavir and ritonavir, LPV/RTV | Abbott Laboratories | 15-Sep-00 | 3.5 months |
| [Lexiva](http://www.accessdata.fda.gov/scripts/cder/drugsatfda/index.cfm?fuseaction=Search.SearchAction&SearchTerm=Lexiva&SearchType=BasicSearch) | Fosamprenavir Calcium, FOS-APV | GlaxoSmithKline | 20-Oct-03 | 10 months |
| [Norvir](http://www.accessdata.fda.gov/scripts/cder/drugsatfda/index.cfm?fuseaction=Search.SearchAction&SearchTerm=Norvir&SearchType=BasicSearch) | ritonavir, RTV | Abbott Laboratories | 1-Mar-96 | 2.3 months |
| [Prezista](http://www.accessdata.fda.gov/scripts/cder/drugsatfda/index.cfm?fuseaction=Search.SearchAction&SearchTerm=Prezista&SearchType=BasicSearch) | darunavir | Tibotec, Inc. | 23-Jun-06 | 6 months |
| [Reyataz](http://www.accessdata.fda.gov/scripts/cder/drugsatfda/index.cfm?fuseaction=Search.SearchAction&SearchTerm=Reyataz&SearchType=BasicSearch) | atazanavir sulfate, ATV | Bristol-Myers Squibb | 20-Jun-03 | 6 months |
| [Viracept](http://www.accessdata.fda.gov/scripts/cder/drugsatfda/index.cfm?fuseaction=Search.SearchAction&SearchTerm=Viracept&SearchType=BasicSearch) | nelfinavir mesylate, NFV | Agouron Pharmaceuticals | 14-Mar-97 | 2.6 months |

**Fusion Inhibitors**

| **Brand Name** | **Generic Name** | **Manufacturer Name** | **Approval Date** | **Time to Approval** |
| --- | --- | --- | --- | --- |
| [Fuzeon](http://www.accessdata.fda.gov/scripts/cder/drugsatfda/index.cfm?fuseaction=Search.SearchAction&SearchTerm=Fuzeon&SearchType=BasicSearch) | enfuvirtide, T-20 | Hoffmann-La Roche & Trimeris | 13-Mar-03 | 6 months |

**Entry Inhibitors - CCR5 co-receptor antagonist**

| **Brand Name** | **Generic Name** | **Manufacturer Name** | **Approval Date** | **Time to Approval** |
| --- | --- | --- | --- | --- |
| [Selzentry](http://www.accessdata.fda.gov/scripts/cder/drugsatfda/index.cfm?fuseaction=Search.SearchAction&SearchTerm=Selzentry&SearchType=BasicSearch) | maraviroc | Pfizer | 06-August-07 | 8 months |

**HIV integrase strand transfer inhibitors**

| **Brand Name** | **Generic Name** | **Manufacturer Name** | **Approval Date** | **Time to Approval** |
| --- | --- | --- | --- | --- |
| [Isentress](http://www.accessdata.fda.gov/scripts/cder/drugsatfda/index.cfm?fuseaction=Search.SearchAction&SearchTerm=Isentress&SearchType=BasicSearch) | raltegravir | Merck & Co., Inc. | 12--Oct-07 | 6 months |

**Figure A1. Basic structure of HIV simulation model.** See text for details

**Figure A2. Typical patient histories with and without pipeline drugs.** See text for details

**Figure A3: The Quantile-Quantile plot for pipeline arrival process**

Quantile-Quantile plots are used to compare a dataset to a theoretical distribution. It provides an assessment of graphical goodness of fit. If the points lie on the line, the probability distribution is acceptable.

**References**

1. Braithwaite RS, Justice AC, Chang CC, Fusco JS, Raffanti SR, Wong JB, et al. Estimating the proportion of patients infected with HIV who will die of comorbid diseases. Am J Med. 2005;118(8):890-8.

2. Braithwaite RS, Nucifora KA, Yiannoutsos CT, Musick B, Kimaiyo S, Diero L, et al. Alternative antiretroviral monitoring strategies for HIV-infected patients in east Africa: opportunities to save more lives? J Int AIDS Soc. 2011;14:38. PMCID: PMC3163507.

3. Braithwaite RS, Roberts MS, Chang CC, Goetz MB, Gibert CL, Rodriguez-Barradas MC, et al. Influence of alternative thresholds for initiating HIV treatment on quality-adjusted life expectancy: a decision model. Ann Intern Med. 2008;148(3):178-85. PMCID: PMC3124094.

4. Braithwaite RS, Shechter S, Chang CC, Schaefer A, Roberts MS. Estimating the rate of accumulating drug resistance mutations in the HIV genome. Value Health. 2007;10(3):204-13.

5. Braithwaite RS, Shechter S, Roberts MS, Schaefer A, Bangsberg DR, Harrigan PR, et al. Explaining variability in the relationship between antiretroviral adherence and HIV mutation accumulation. J Antimicrob Chemother. 2006;58(5):1036-43.

6. Johnson VA, Brun-Vezinet F, Clotet B, Gunthard HF, Kuritzkes DR, Pillay D, et al. Update of the drug resistance mutations in HIV-1: December 2010. Top HIV Med. 2010;18(5):156-63.
