# Supplementary figures and images for: Correction: Should Expectations about the Rate of New Antiretroviral Drug Development Impact the Timing of HIV Treatment Initiation and Expectations about Treatment Benefits?
Source: PLoS One. 2014 Sep 12;9(9):e108643. doi: 10.1371/journal.pone.0108643 (PMC4162648; doi:10.1371/journal.pone.0108643)

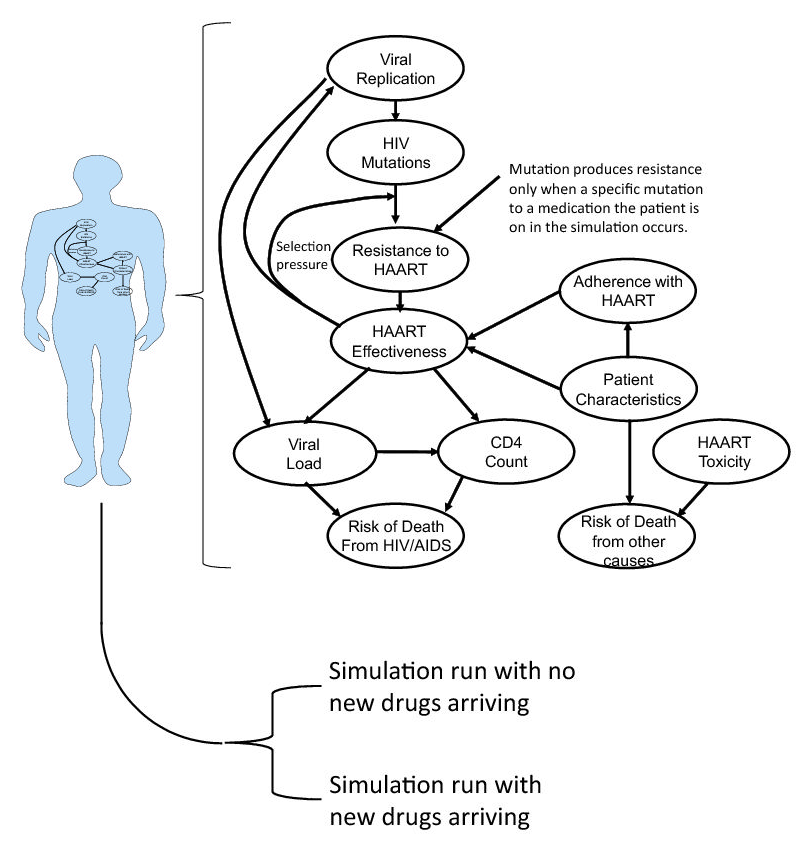

Supplement: Figure S1 — Basic structure of HIV simulation model.See text for details. (TIF) [file pone.0108643.s001.tif]

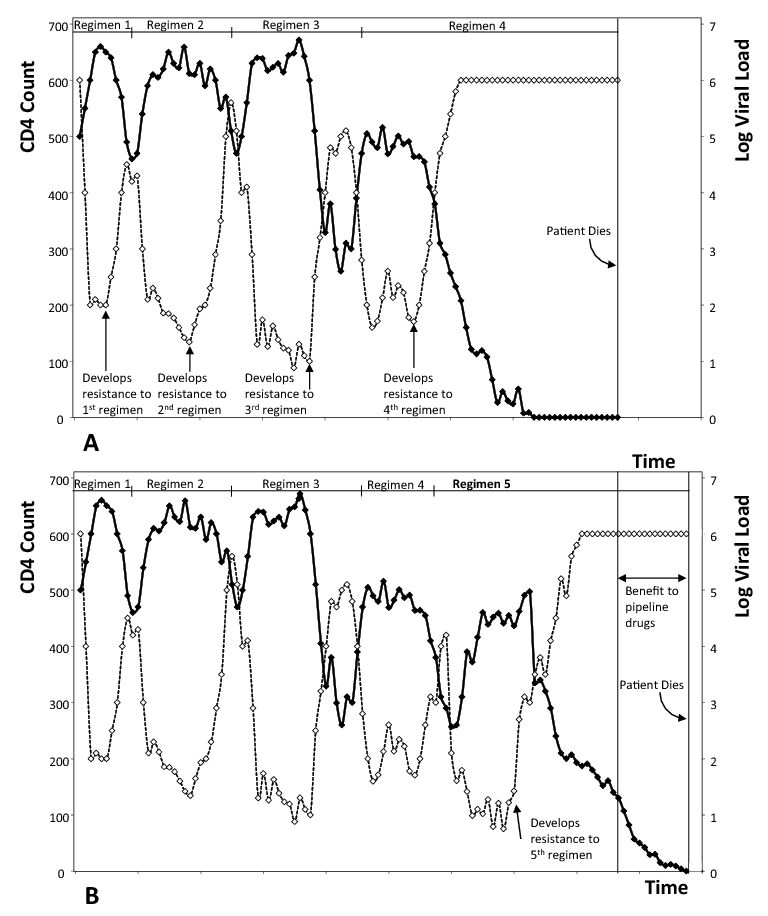

Supplement: Figure S2 — Typical patient histories with and without pipeline drugs. See text for details. (TIF) [file pone.0108643.s002.tif]

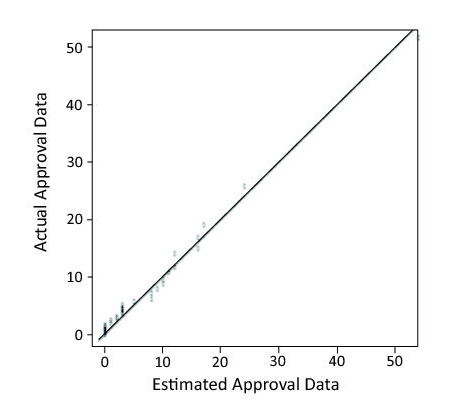

Supplement: Figure S3 — The Quantile-Quantile plot for pipeline arrival process. Quantile-Quantile plots are used to compare a dataset to a theoretical distribution. It provides an assessment of graphical goodness of fit. If the points lie on the line, the probability distribution is acceptable. (TIF) [file pone.0108643.s003.tif]
